# Comparison of polysomnographic and cephalometric parameters based on positional and rapid eye movement sleep dependency in obstructive sleep apnea

**DOI:** 10.1038/s41598-022-13850-6

**Published:** 2022-06-14

**Authors:** Jung-Hwan Jo, Sung-Hun Kim, Ji-Hee Jang, Ji-Woon Park, Jin-Woo Chung

**Affiliations:** 1 Department of Oral Medicine, Seoul National University Dental Hospital, 101 Daehak-ro, Jongno-gu, Seoul, 03080 Korea; 2 Department of Oral Medicine and Oral Diagnosis, School of Dentistry and Dental Research Institute, Seoul National University, 101 Daehak-ro, Jongno-gu, Seoul, 03080 Korea

**Keywords:** Sleep disorders, Respiratory signs and symptoms

## Abstract

The aim of this study is to investigate the differences in polysomnographic and cephalometric features according to positional and rapid eye movement (REM) sleep dependencies in obstructive sleep apnea patients. Standard polysomnography and cephalometric analyses were performed on 133 OSA patients. The subjects were categorized into positional and non-positional, and REM-related and not-REM-related OSA groups according to positional and REM sleep dependency on severity of sleep apnea. Polysomnographic and cephalometric parameters were compared between groups. Positional and REM-related OSA patients showed significantly lower non-supine apnea–hypopnea index (AHI), non-REM (NREM) AHI and overall AHI and higher NREM oxygen saturation (SpO_2_) and mean SpO_2_ compared to non-positional and not-REM-related OSA patients, respectively. Cephalometric features between positional and non-positional OSA patients did not show any significant differences. However, REM-related OSA patients showed significantly larger inferior oral airway space and shorter perpendicular distance between mandibular plane and anterior hyoid bone and the distance between uvula and posterior nasal spine, and narrower maximum width of soft palate than not-REM-related OSA patients. Positional and REM-related OSA patients have lower severity of sleep apnea, suggesting the possibility of lower collapsibility of the upper airway. REM sleep dependency was associated with anatomical factors, while positional dependency did not show such a tendency.

## Introduction

Obstructive sleep apnea (OSA) is a common medical condition characterized by repetitive occlusions of the upper airway during sleep. It is known to disturb normal sleep architecture and cause intermittent hypoxia that is associated with daytime sleepiness, and increased incidence of cardiovascular, neuropsychiatric, and endocrinologic disorders^1–3^. It is well known that factors such as older age, male sex, higher body mass index (BMI) and alcohol and sedative drug intake worsen OSA severity^4^.

In addition to the well-known risk factors of OSA, an increasing amount of literature has been published on the role of body position during sleep in OSA and efficient approaches to avoid sleeping positions that worsen OSA severity^5^. Patients with OSA have been repeatedly reported to have more obstructive events in the supine position than in other non-supine positions, and not only the frequency but also the duration of apneas are influenced by body position as well^6^. Such observations have led to the definition of the so-called positional OSA. Positional OSA patients are defined as those who have a supine respiratory disturbance index (RDI) or apnea–hypopnea index (AHI) at least twice the value compared to that observed in non-supine sleeping positions^7^. Positional OSA patients are generally known to be younger and less obese implicating the possibility that such patients may have a less severe phenotype of respiratory disturbance than the non-positional patients. Non-positional OSA patients show higher AHI and lower mean oxygen saturation (SpO_2_) levels compared to positional OSA patients^8^. However other studies reported that there were no significant differences in clinical characteristics such as BMI between positional and non-positional OSA patients^9^. Positional therapy changing to the non-supine position during sleep can be a simple and effective measure in reducing AHI, but unsatisfactory for patients with non-positional OSA, emphasizing the need of further related investigations for accurate diagnosis^10^.

Another phenotype that can be identified in OSA patients is based on the rapid eye movement (REM) relatedness of respiratory events. Upper airway muscle activation which is reduced during REM sleep rather than during non-rapid eye movement (NREM) sleep, resulting in increased apnea and consequent hypoxemia. REM-related OSA is defined as REM AHIs that are at least two times higher than NREM AHIs^11^. REM predominant OSA is well known as a result of muscle atonia, but the mechanism of NREM predominant OSA has not yet been fully elucidated^12^. Patients with REM-related OSA are known to show characteristics that differ from the general features of OSA as it is more commonly observed in younger age, women, and less severe OSA patients^11^. In spite of the fact that both positional and REM-related OSA are known to be associated with treatment effectiveness, current literature lacks sufficient evidence to their mechanism and clinical implications regarding predictors of treatment success^13,14^.

Therefore, we analyzed the relationship between risk factors of OSA and anatomic factors of the upper airway based on polysomnographic and cephalometric measures according to the positional and REM sleep dependencies in patients with OSA to investigate their inter-relationship with clinical characteristics and provide diagnostic guidelines.

## Results

### Demographic features

Among the 133 subjects, 113 (85.0%) were men and 20 (15.0%) were women. Mean age was 44.0 ± 12.3 years (range 20–82 years). Fifty-four patients had mild, 32 moderate, and 47 severe OSA. Mean values of BMI, neck circumference, and ESS were 25.3 ± 3.4 kg/m^2^, 37.1 ± 6.3 cm, and 8.3 ± 4.3, respectively.

When comparing age, gender, BMI, neck circumference, and ESS according to positional dependency and REM dependency, non-positional OSA patients showed a significantly higher BMI compared to positional OSA patients, and REM-related OSA patients were significantly younger than not-REM-related OSA patients (Table 1). Gender, neck circumferences, and ESS between groups did not show any significant differences.

**Table 1:** Demographic features according to positional and REM dependency.

Variable	Positional dependency			REM dependency		
	Positional (n = 111)	Non-positional (n = 22)	P value	REM-related (n = 53)	Not-REM-related (n = 80)	P value
Age (years)^a^	43.5 ± 12.9	46.2 ± 8.9	0.349	40.1 ± 11.0	46.5 ± 12.5	0.003*
Age group (> 55 years old)^b^	87.5%	12.5%	0.764	25.0%	75.0%	0.101
Gender (male-to-female ratio)^b^	83.8%	90.9%	0.526	81.1%	87.5%	0.314
BMI (kg/m^2^)^a^	24.9 ± 3.3	27.2 ± 3.1	0.003*	25.5 ± 3.3	25.1 ± 3.5	0.565
Neck circumference (cm)^a^	36.6 ± 6.7	39.7 ± 2.9	0.074	37.2 ± 3.8	37.0 ± 7.4	0.911
ESS^a^	8.5 ± 4.5	7.3 ± 3.3	0.255	8.0 ± 3.6	8.5 ± 4.7	0.565
ESS > 10^b^	93.3%	6.7%	0.081	36.7%	63.3%	0.759

REM, rapid eye movement sleep; BMI, body mass index; ESS, Epworth sleepiness scale.

^a^Results were obtained from independent T-test: mean ± SD.

^b^Results were obtained from Chi-square test.

*Significant difference: p < 0.05.

### Polysomnographic characteristics

Positional OSA patients showed significantly higher total sleep time and a lower REM arousal index, NREM arousal index, and respiratory arousal index than non-positional OSA patients (Table 2). REM-related OSA patients showed significantly higher sleep efficiency, NREM stage II sleep, REM sleep, and percentage time of supine position during sleep, while sleep latency, NREM stage I sleep, NREM arousal index, respiratory arousal index, and total arousal index was lower compared to not-REM-related OSA patients.

**Table 2:** Polysomnographic characteristics according to positional and REM dependency-sleep architecture.

Variable	Positional dependency			REM dependency		
	Positional (n = 111)	Non-positional (n = 22)	P value	REM-related (n = 53)	Not-REM-related (n = 80)	P value
Total sleep time (mins)	326.8 ± 62.0	290.3 ± 66.1	0.014*	330.2 ± 53.9	314.5 ± 69.4	0.165
Sleep efficiency (%TST)	81.6 ± 10.7	75.7 ± 15.9	0.106	83.5 ± 10.2	78.7 ± 12.5	0.016*
Sleep latency (mins)	15.1 ± 14.3	21.2 ± 24.5	0.110	12.7 ± 10.6	18.3 ± 19.1	0.032*
REM latency (mins)	118.0 ± 56.5	124.3 ± 70.4	0.647	123.9 ± 59.8	115.8 ± 58.3	0.438
Sleep stage I (%TST)	26.8 ± 12.4	34.4 ± 25.5	0.187	18.7 ± 7.6	34.3 ± 16.2	< 0.001*
Sleep stage II (%TST)	48.8 ± 12.6	44.6 ± 21.1	0.368	55.1 ± 11.4	43.5 ± 14.3	< 0.001*
Sleep stage III + IV (%TST)	2.8 ± 5.7	1.2 ± 2.7	0.219	3.3 ± 5.2	2.0 ± 5.4	0.172
REM sleep (%TST)	17.4 ± 6.8	14.3 ± 7.1	0.058	18.3 ± 6.7	15.9 ± 6.9	0.042*
Time of supine position (% TST)	72.1 ± 25.2	74.1 ± 22.5	0.720	77.8 ± 23.8	68.8 ± 24.8	0.040*
REM arousal index	15.0 ± 13.2	25.9 ± 17.3	0.013*	17.5 ± 12.4	16.3 ± 15.8	0.644
NREM arousal index	12.7 ± 13.0	25.1 ± 26.5	0.049*	5.9 ± 5.5	21.1 ± 18.8	< 0.001*
Respiratory arousal index	15.2 ± 13.5	30.2 ± 28.4	0.023*	8.6 ± 7.0	23.6 ± 20.0	< 0.001*
Snoring arousal index	4.4 ± 3.6	3.2 ± 3.1	0.139	4.6 ± 3.5	3.9 ± 3.6	0.326
Spontaneous arousal index	3.9 ± 3.4	2.9 ± 3.0	0.219	3.4 ± 3.1	3.9 ± 3.6	0.397
Total arousal index	28.1 ± 12.8	38.5 ± 25.9	0.080	21.3 ± 8.6	35.5 ± 17.4	< 0.001*

REM, rapid eye movement sleep; TST, total sleep time; NREM, non-rapid eye movement sleep.

^a^Results were obtained from independent T-test: mean ± SD.

*Significant difference: p < 0.05.

Table 3 shows the respiratory parameters and oxygen saturation levels according to positional and REM dependency. Positional OSA patients showed significantly lower non-supine AHI, REM AHI, NREM AHI, and overall AHI compared to non-positional OSA patients. There was no significant difference in the supine AHI according to positional dependency. REM-related OSA patients showed significantly lower supine AHI, non-supine AHI, NREM AHI, and overall AHI, while REM AHI and percentage of severe OSA patients was higher compared to not-REM-related OSA patients.

**Table 3:** Polysomnographic characteristics according to positional and REM dependency- respiratory parameters.

Variable	Positional dependency			REM dependency		
	Positional (n = 111)	Non-positional (n = 22)	P value	REM-related (n = 53)	Not-REM-related (n = 80)	P value
Total AHI^a^	24.9 ± 18.2	43.1 ± 36.3	0.031*	17.2 ± 11.0	35.0 ± 26.2	< 0.001*
Supine AHI^a^	33.3 ± 23.4	44.9 ± 37.7	0.177	20.7 ± 14.9	44.8 ± 28.1	< 0.001*
Non-supine AHI^a^	6.0 ± 8.8	38.9 ± 35.6	< 0.001*	6.0 ± 8.4	15.0 ± 24.9	0.004*
REM AHI^a^	33.1 ± 21.8	44.5 ± 28.6	0.036*	40.7 ± 18.4	31.2 ± 25.5	0.014*
NREM AHI^a^	23.0 ± 19.4	42.6 ± 38.2	0.028*	12.0 ± 9.3	35.7 ± 26.8	< 0.001*
Severity group^b^	32.4%	50.0%	0.115	13.2%	50.0%	< 0.001*
Mean SpO_2_^a^	95.4 ± 1.7	94.1 ± 2.5	0.025*	95.8 ± 1.6	94.8 ± 2.0	0.004*
Lowest SpO_2_^a^	81.3 ± 9.2	77.1 ± 11.3	0.061	83.2 ± 7.3	78.8 ± 10.6	0.005*
Time below 90% SpO_2_ (%)^a^	4.0 ± 7.0	10.2 ± 14.2	0.059	1.6 ± 2.6	7.3 ± 10.7	0.000*
REM SpO_2_^a^	94.2 ± 9.4	92.7 ± 3.8	0.476	95.3 ± 1.9	93.1 ± 11.1	0.154
NREM SpO_2_^a^	95.4 ± 1.7	94.0 ± 2.8	0.028*	95.8 ± 1.6	94.8 ± 2.1	0.005*

REM, rapid eye movement sleep; AHI, apnea–hypopnea index; NREM, non-rapid eye movement sleep.

^a^Results were obtained from independent T-test: mean ± SD.

^b^Results were obtained from Chi-square test.

*Significant difference: p < 0.05.

Positional OSA patients showed higher mean SpO_2_, NREM SpO_2_, lowest SpO_2_ and REM SpO_2_, and lower percentage of time below 90% SpO_2_ than non-positional OSA patients, but the differences reached statistical significance only for mean SpO_2_ and NREM SpO_2_ (p < 0.05). Not-REM-related OSA patients showed significantly lower mean SpO_2_, lowest SpO_2_ and NREM SpO_2_, and higher percentage of time below 90% SpO_2_ compared to REM-related OSA patients (p < 0.01).

### Cephalometric parameters according to positional dependency and REM dependency

Table 4 shows cephalometric analyses of 12 linear and angular variables. Cephalometric parameters between positional and non-positional OSA patients did not show any significant differences. However, REM-related OSA patients showed significantly larger inferior oral airway space and shorter perpendicular distance between anterior hyoid bone and mandibular plane and the distance between uvula and posterior nasal spine, and narrower maximum width of soft palate than not-REM-related OSA patients as shown in Table 5.

**Table 4:** Cephalometric parameters according to positional dependency.

Variable	Positional (n = 111)	Non-positional (n = 22)	P value
SNA	79.0 ± 4.9	80.2 ± 5.7	0.322
ANB	0.4 ± 3.2	− 0.6 ± 3.5	0.187
AH⊥MP	20.5 ± 10.6	22.6 ± 6.1	0.367
AH-C3	42.9 ± 6.8	44.0 ± 5.0	0.479
SPT	12.2 ± 4.6	12.6 ± 2.3	0.748
PNS-U	40.7 ± 5.8	41.3 ± 4.6	0.635
NL/PNS-U	83.0 ± 34.2	84.4 ± 27.6	0.859
NAS	25.7 ± 3.6	24.7 ± 3.8	0.245
SOAS	11.8 ± 3.6	11.2 ± 3.2	0.485
MOAS	9.1 ± 3.5	8.9 ± 3.4	0.815
IOAS	11.1 ± 3.7	10.4 ± 3.8	0.389
HAS	18.2 ± 5.7	17.2 ± 4.8	0.421

SNA, smaller angle which is formed by sella (S), nasion (N), and A point; ANB, smaller angle which is formed by A point, nasion (N), and B point; AH⊥MP, the perpendicular distance between AH and Go-Me line; AH-C3, the distance between AH and the third vertebra; SPT, maximum width of soft palate which is perpendicular to PNS-U line; PNS-U, the distance between PNS and U; NL/PNS-U, smaller angle between ANS-Pm line and PNS-U line; NAS, nasal airway space; SOAS, superior oral airway space; MOAS, middle oral airway space; IOAS, inferior oral airway space; HAS, hyoid airway space.

Results were obtained from independent T test: mean ± SD.

**Table 5:** Cephalometric parameters according to REM dependency.

Variable	REM-related (n = 53)	Not-REM-related (n = 80)	P value
SNA	79.1 ± 5.8	79.3 ± 4.5	0.800
ANB	− 0.2 ± 3.0	0.6 ± 3.4	0.146
AH⊥MP	18.4 ± 5.5	22.5 ± 11.8	0.019*
AH-C3	42.7 ± 5.2	43.4 ± 7.2	0.537
SPT	11.3 ± 2.3	12.9 ± 5.1	0.031*
PNS-U	39.6 ± 4.8	41.6 ± 5.9	0.045*
NL/PNS-U	84.9 ± 35.6	82.2 ± 31.6	0.648
NAS	25.7 ± 4.0	25.4 ± 3.4	0.696
SOAS	12.3 ± 3.5	11.2 ± 3.5	0.077
MOAS	9.5 ± 3.4	8.7 ± 3.4	0.197
IOAS	12.1 ± 4.2	10.3 ± 3.2	0.006*
HAS	18.5 ± 5.6	17.8 ± 5.5	0.490

REM, rapid eye movement sleep; SNA, smaller angle which is formed by sella (S), nasion (N), and A point; ANB, smaller angle which is formed by A point, nasion (N), and B point; AH⊥MP, the perpendicular distance between AH and Go-Me line; AH-C3, the distance between AH and the third vertebra; SPT, maximum width of soft palate which is perpendicular to PNS-U line; PNS-U, the distance between PNS and U; NL/PNS-U, smaller angle between ANS-Pm line and PNS-U line; NAS, nasal airway space; SOAS, superior oral airway space; MOAS, middle oral airway space; IOAS, inferior oral airway space; HAS, hyoid airway space.

Results were obtained from independent T-test: mean ± SD.

*Significant difference: p < 0.05.

### Correlations of polysomnographic and cephalometric parameters

Pearson’s correlation analysis was carried out between polysomnographic and cephalometric parameters. BMI was significantly correlated with all polysomnographic variables except time of supine position. Age and neck circumference were significantly correlated with total AHI, supine AHI, non-supine AHI, and NREM AHI. AH-C3 and PNS-U were significantly correlated with mean SpO_2_, lowest SpO_2,_ NREM SpO_2_, supine AHI and NREM AHI. AH⊥MP was significantly correlated with total AHI, supine AHI, NREM AHI and time below 90% SpO_2_. IOAS was significantly correlated with total AHI and NREM AHI.

### Regression analysis of risk factors on positional dependency and REM dependency

Logistic regression analysis results showed that only BMI had a significant effect on positional dependency (p = 0.004, β = − 0.227) (Table 6).

**Table 6:** Logistic regression analysis of the risk factors on positional dependency.

Predictor variables	Standardized β	Standard error	Odds ratio	95% CI	P value
Age	− 0.018	0.019	0.982	0.946–1.020	0.347
Gender (men = 1, women = 2)	− 0.660	0.785	0.517	0.111–2.407	0.400
BMI	− 0.227	0.079	0.797	0.683–0.930	0.004*
Severe OSA	− 0.734	0.472	0.480	0.190–1.211	0.120
SNA	− 0.047	0.047	0.954	0.870–1.047	0.321
ANB	0.096	0.073	1.101	0.954–1.270	0.188
AH⊥MP	− 0.017	0.019	0.984	0.947–1.021	0.389
AH-C3	− 0.026	0.036	0.975	0.908–1.046	0.476
SPT	− 0.016	0.049	0.984	0.894–1.083	0.747
PNS-U	0.020	0.043	0.980	0.901–1.065	0.632
NL/PNS-U	− 0.001	0.007	0.999	0.985–1.013	0.858
NAS	0.076	0.065	1.079	0.949–1.227	0.244
SOAS	0.048	0.069	1.049	0.917–1.200	0.483
MOAS	0.016	0.069	1.016	0.888–1.163	0.814
IOAS	0.058	0.067	1.060	0.929–1.210	0.386
HAS	0.036	0.044	1.036	0.951–1.129	0.418

CI, confidence interval; BMI, body mass index; severe OSA, obstructive sleep apnea showing apnea–hypopnea index ≥ 30; SNA, smaller angle which is formed by sella (S), nasion (N), and A point; ANB, smaller angle which is formed by A point, nasion (N), and B point; AH⊥MP, the perpendicular distance between AH and Go-Me line; AH-C3, the distance between AH and the third vertebra; SPT, maximum width of soft palate which is perpendicular to PNS-U line; PNS-U, the distance between PNS and U; NL/PNS-U, smaller angle between ANS-Pm line and PNS-U line; NAS, nasal airway space; SOAS, superior oral airway space; MOAS, middle oral airway space; IOAS, inferior oral airway space; HAS, hyoid airway space.

*Significant difference: p < 0.05.

Factors including age (p = 0.004, β = − 0.046), having severe OSA (p < 0.001, β = − 1.883), the perpendicular distance between AH and Go-Me line (AH⊥MP; p = 0.007, β = − 0.085), maximum width of soft palate which is perpendicular to PNS-U line (SPT; p = 0.019, β = − 0.176) and inferior oral airway space (p = 0.009, β = 0.136) had a significant effect on REM dependency (Table 7).

**Table 7:** Logistic regression analysis of the risk factors on REM dependency.

Predictor variables	Standardized β	Standard error	Odds ratio	95% CI	P value
Age	− 0.046	0.016	0.955	0.926–0.986	0.004*
Gender (men = 1, women = 2)	− 0.487	0.487	0.614	0.236–1.597	0.317
BMI	0.031	0.053	1.031	0.929–1.145	0.562
Severe OSA	− 1.883	0.463	0.152	0.061–0.377	< 0.001*
SNA	− 0.009	0.036	0.991	0.924–1.062	0.798
ANB	− 0.081	0.056	0.922	0.826–1.029	0.147
AH⊥MP	− 0.085	0.031	0.919	0.864–0.977	0.007*
AH-C3	− 0.017	0.028	0.983	0.931–1.038	0.534
SPT	− 0.176	0.075	0.839	0.724–0.971	0.019*
PNS-U	− 0.066	0.034	0.936	0.876–1.000	0.050
NL/PNS-U	0.002	0.005	1.002	0.992–1.013	0.646
NAS	0.019	0.049	1.020	0.926–1.123	0.694
SOAS	0.090	0.051	1.094	0.989–1.210	0.080
MOAS	0.068	0.053	1.070	0.965–1.186	0.197
IOAS	0.136	0.052	1.146	1.034–1.269	0.009*
HAS	0.022	0.032	1.023	0.960–1.089	0.488

REM, rapid eye movement sleep; CI, confidence interval; BMI, body mass index; severe OSA, obstructive sleep apnea showing apnea–hypopnea index ≥ 30; SNA, smaller angle which is formed by sella(S), nasion(N), and A point; ANB, smaller angle which is formed by A point, nasion(N), and B point; AH⊥MP, the perpendicular distance between AH and Go-Me line; AH-C3, the distance between AH and the third vertebra; SPT, maximum width of soft palate which is perpendicular to PNS-U line; PNS-U, the distance between PNS and U; NL/PNS-U, smaller angle between ANS-Pm line and PNS-U line; NAS, nasal airway space; SOAS, superior oral airway space; MOAS, middle oral airway space; IOAS, inferior oral airway space; HAS, hyoid airway space.

*Significant difference: p < 0.05.

## Discussion

This is the first study to analyze polysomnographic features, cephalometric parameters and contributors on both positional and REM dependency in OSA patients. The results showed that positional and REM-related OSA patients had more mild traits of OSA compared to non-positional and not-REM-related OSA patients, respectively. Anatomical factors were more closely related to REM dependency than positional dependency.

Previous studies have reported that positional OSA patients had less severe overall AHI, higher oxygen saturation, lower percentage of time below 90% oxygen saturation than non-positional OSA patients^9,15^. REM-related OSA was usually common in younger age and less severe cases than not-REM-related OSA^11,16^. The results of this study were consistent with previous literature. It is generally accepted that supine position during sleep has detrimental effects on sleep breathing disorder symptoms. Most previous studies on the effect of body position on sleep apnea have shown that sleeping in a supine position increases the severity of sleep apnea^15,17^. Some researchers suggested that positional OSA patients appear to have a milder form of OSA because such patient spend less sleep time in the supine position, implying the possibility of successfully applying positional therapy, but its efficacy remains controversial^18,19^. There was no significant difference in time spent in supine position between positional and non-positional OSA patients of our study. The underlying mechanism may rather be related to obesity. In this study, positional OSA patients had a significantly lower BMI compared to non-positional OSA patients, and logistic regression analysis results showed that BMI was the only significant risk factor for positional dependency. Weight gain can result in an increase in the thickness of the lateral pharyngeal walls of the upper airway which are already narrow in OSA patients. It can also result in further narrowing of the lumen and increase in collapsibility of pharyngeal space even in a lateral sleeping position^20^. Another relevant hypothesis is that positional OSA is an intermediate state in the progression from snoring to OSA^18^. Recent researches on the interaction between unfavorable upper airway geometry, reduced lung volume, instability of upper airway dilator muscles, arousal threshold, and ventilatory control instability have improved our understanding on the effect of positional dependency on upper airway collapsibility^21^.

In accordance with previous studies, REM-related OSA was more commonly observed in younger age, women, and had less severe apnea symptoms compared to not-REM-related OSA patients in our study^11,22^. The results of our study also showed that REM-related OSA patients were younger, and had higher sleep efficiency and lower overall arousal index than non-REM-related OSA patients. Moreover, not-REM-related OSA patients showed significantly higher supine AHI, overall AHI, non-supine AHI, NREM AHI, and lower mean oxygen saturation and NREM oxygen saturation compared to REM-related OSA patients. Several possible mechanisms for REM dependency have been proposed until now. Muscle tone of the tongue and pharyngeal dilator muscles decrease and the upper respiratory resistance increases in REM sleep, so that OSA appears more easily and in a more severe level in REM sleep than in NREM sleep^23^. Meanwhile, NREM predominant OSA was suggested to be associated with ventilatory instability, which is a cause of greater dynamic reduction in ventilation before and after wakefulness^24^.

The results of this study showed that the prevalence of OSA was higher in men (85.0%) than in women, and the male-to-female ratio was lower in positional and REM-related OSA patients than non-positional and not-REM-related OSA patients, respectively, but the difference was not statistically significant. Such findings are consistent with previous studies reporting male predominance, especially a higher male-to female ratio in more severe OSA groups^25^. The prevalence of positional OSA was 83.5% in our study. This value is somewhat higher than that reported in previous studies which report a prevalence of 53 to 72% in OSA patients and higher in the Asian population compared to Caucasians^10,26^. The difference might be due to the tendency that Asians have less obese than whites. Because obesity was one of the most important factors for positional dependency as shown in this study, Asians with lower BMI value tend to have the higher prevalence of positional OSA.

Previous studies found that positional OSA patients with higher non-supine AHI tend to more easily transform into non-positional OSA patients within a few years^20,26^. This implies the importance of early diagnosis and intervention of positional OSA patients to prevent progression into a more severe OSA type. Identification of positional dependency could be important for diagnosis as well as in the evaluation of treatment efficacy. Such factors might be relevant when choosing treatment modalities including positional therapy, continuous airway positive pressure (CPAP), and mandibular advancement devices (MAD), since the latter was found to be effective in positional OSA patients. Furthermore, it has been reported that the combination of CPAP, MAD, and positional therapy is more effective than applying any one treatment modality alone^27^.

Interestingly, logistic regression analysis revealed age, severe OSA, and several anatomical variables as risk factors for REM dependency, but not gender and BMI. The results on the association with age, severe OSA, and BMI on REM dependency generally are in line with those from prior studies^11,28^. However, the role of gender on REM dependency is less clear. Several studies explained female predominance in REM-related OSA patients which could be explained by gender differences in upper airway stability and hormonal factors. It is known that men show greater upper airway resistance and collapsibility in NREM sleep than women, and women have greater genioglossus activity in the waking state than men^29,30^. In contrast, others showed that there were no associations between gender and REM dependency^16^.

Although certain craniofacial structures are generally known as risk factors for OSA, there were few studies about cephalometric analysis results in REM-related OSA patients^13^. Therefore, the results of this study may provide clinical evidences on the pathophysiological role of craniofacial characteristics in REM-related OSA. An inferiorly displaced hyoid bone as measured by AH⊥MP, large soft plate measured by SPT and inferior oral airway space were shown as risk factors for REM dependency.

REM dependency may be affected by unfavorable conditions of the hard and soft tissue surrounding oral and upper airway space, and reflect unique features that are different from those related to positional dependency.

There are several limitations in our study. First, this study was limited by its retrospective design. Confounding factors that may affect positional and REM dependency may have been neglected due to bias in subject and data selection. Nevertheless, we collected and analyzed all clinical, polysomnographic, and cephalometric data from consecutive patients following strict selection criteria to lessen the possibility of bias. Second, lateral cephalometric radiographs were taken in upright position under wakefulness. Although this is due to an inherent limitation of the way in which standardized lateral cephalometric radiographs are taken, it cannot truly reflect the positional characteristics of the hard and soft tissue during sleep. Magnetic resonance imaging under sedation could be considered as an alternative, but it is difficult to implement such a method to all patients due to high cost and time related issues. Further extensive studies based on various clinical variables and treatment outcomes are needed to more comprehensively understand the exact pathophysiology of positional and REM-related OSA.

## Methods

### Subjects

Level 1 standard polysomnography and radiographic lateral cephalometric analysis data of consecutive patients who visited the Snoring and Sleep Apnea Clinic, Department of Oral Medicine, Seoul National University Dental Hospital complaining of snoring, sleep apnea, and related symptoms including excessive daytime sleepiness from June, 2006 to April, 2016 were retrospectively evaluated. Among a total of 251 patients, 170 patients were diagnosed as OSA based on an apnea–hypopnea index (AHI) ≥ 5. Among the OSA patients, criteria for exclusion were severe uncontrolled cardiovascular disease, active psychiatric disease, seizure disorders, medication usage for sleeping disorders, head and neck injuries, history of major surgery on the orofacial region, pregnancy, and concomitant sleep disorders that could affect the sleep study results. Also, those under the age of 20 years and missing cephalometric analysis data were excluded. The final analysis included 133 patients, consisted of 113 men and 20 women, aged from 20 to 82 years (mean age 44.0 ± 12.3 years). The study was approved by the Institutional Review Board of Seoul National University Dental Hospital and informed consent was obtained from all individual participants included in the study (CRI14037 and CRI20004). All methods were performed in accordance with the relevant guidelines and regulations by including a statement.

### Polysomnographic evaluation

Overnight multi-channel level 1 polysomnography was performed with Alice 5 (Respironics, Pittsburgh, USA). Signals included electroencephalogram (EEG), both electrooculograms (EOG), electrocardiogram (ECG), submental and tibial electromyogram (EMG), oxygen saturation, nasal cannula, nasal pressure transducer, thoracic and abdominal inductive plethysmography. Body position was monitored via infrared video camera for direct observation of the patient by the technician as well as digital recording through a posture tag on the thoracic piezoelectric belt.

Sleep staging and respiratory events were scored by an eligible sleep specialist according to the standard criteria of the American Academy of Sleep Medicine^31^. Obstructive apneas were defined as a reduction in airflow greater than 90% with a duration of at least 10 s in which there was persistent respiratory effort, whereas hypopneas were defined as a reduction of airflow by 30–90% for more than 10 s accompanied by an oxygen desaturation ≥ 3%.

Daytime sleepiness was examined using the Epworth Sleepiness Scale (ESS) questionnaire. An ESS value more than 10 was considered as having excessive daytime sleepiness^32^. Weight and height were measured and body mass index (BMI) was calculated as weight in kilograms divided by the square of height in meters.

Participants were categorized into positional and non-positional OSA patients following the criteria suggested by Cartwright^17^. Positional OSA patients were defined as those showing a supine AHI at least two times higher than their lateral AHI (supine AHI/non-supine AHI ≥ 2), and non-positional patients were those with a supine AHI less than two times their lateral AHI (supine AHI/non-supine AHI < 2).

Additionally, the participants were categorized as REM-related and not-REM-related OSA according to the REM sleep dependency of OSA severity. REM-related OSA patients were defined as those showing a REM AHI at least two times higher than their NREM AHI (REM AHI/NREM AHI ≥ 2), and not-REM-related patients were those with a REM AHI less than two times their NREM AHI (REM AHI/NREM AHI < 2)^11^.

### Cephalometric analyses

**Figure 1:**
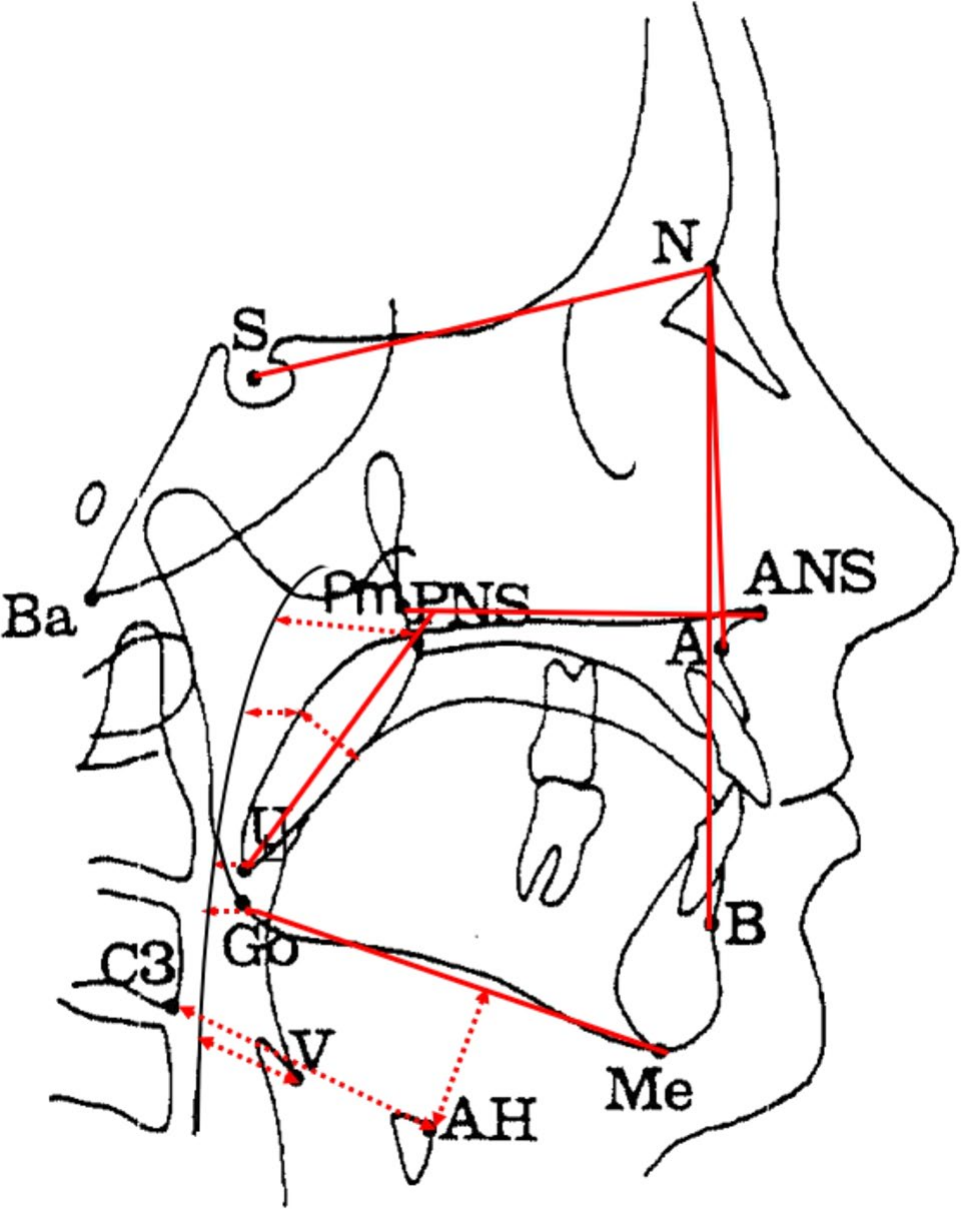
Cephalometric landmarks and linear and angular measurements. S, Sella; N, Nasion; A, subspinale; B, supramentale; AH, Anterior Hyoid; Go, Gonion; Me, Menton; C3, Third Vertebra; PNS, posterior nasal spine; U, Tip of the Uvula; ANS, anterior nasal spine; Pm, Pterygomaxillare; Ba, Basion; V, Vallecula. SNA, smaller angle which is formed by sella(S), nasion(N), and A point; ANB, smaller angle which is formed by A point, nasion(N), and B point. AH⊥MP, the perpendicular distance between AH and Go-Me line; AH-C3, the distance between AH and the third vertebra; SPT, maximum width of soft palate which is perpendicular to PNS-U line; PNS-U, the distance between PNS and U; NL/PNS-U, smaller angle between ANS-Pm line and PNS-U line; NAS, nasal airway space, the distance between the intersection of Ba-PNS and posterior pharyngeal wall and PNS; SOAS, superior oral airway space, the distance between the ventral surface of the soft palate and the dorsal surface of the tongue, measured through a point midway between PNS and U, parallel to the line that intersects gonion and menton; MOAS, middle oral airway space, the perpendicular distance between U and posterior pharyngeal wall; IOAS, inferior oral airway space, the distance between the posterior pharyngeal wall and the dorsal surface of the base of the tongue, measured on the line that intersects gonion and menton; HAS, hyoid airway space, the perpendicular distance between V and posterior pharyngeal wall.

Standardized lateral cephalometric radiographs were taken with Asahi CX-90 SP II (Asahi, Toshiba, Japan) and 10 × 12-in. FCR IP cassette (Fujifilm, Tokyo, Japan). The distance from the anode to midsagittal plane of the patient was 150 cm, while the distance from the midsagittal plane to IP cassette was 15 cm. The magnification factors of the images from the X-ray machine were corrected. Radiographs were taken with the subjects standing, the head fixed with ear rods and a support on the forehead, the teeth in the centric occlusion position, the lips in a relaxed position, and the head in the natural position with the sagittal plane parallel to the film at the end of expiration without swallowing. Cephalometric tracings were performed by an examiner blind to the PSG and clinical examination results using the digitalized V-ceph program (version 5.3, Osstem Inc., Seoul, Korea) to calculate all parameter concerning measures of angles and distances. Twelve variables of linear and angular measurements were calculated from fourteen landmarks digitized on each radiograph based on the methods of Kirjavainen analysis as described in Fig. 1^33^.

### Statistical analyses

Differences between positional and non-positional or between REM-related and not-REM-related OSA groups in demographic and clinical features (age, age groups, BMI, ESS scores and ESS scores group), and polysomnographic features (total sleep time, sleep stage, percent time of supine position, REM during sleep, AHIs, oxygen saturation during sleep, and OSA severity groups) were analyzed with the independent t test and Chi-square test. Results of cephalometric analyses of the groups were also analyzed with independent t test. Correlations of polysomnographic and cephalometric parameters were analyzed by Pearson’s correlation coefficient. Factors associated with positional and REM dependency were analyzed through logistic regression analysis.

## Conclusions

The results of our study suggested that positional and REM-related OSA patients have a less collapsible airway and showed lower severity of sleep apnea compared to non-positional and not-REM-related OSA patients, respectively. REM sleep dependency was associated with anatomical factors, while positional dependency did not show such a tendency. The findings may guide clinicians in defining and characterizing the patient for optimal treatment planning.

## Data Availability

The datasets generated during and/or analyzed during the current study are available from the corresponding author on reasonable request.

## References

[1] Kapur VK, Resnick HE, Gottlieb DJ, Sleep Heart Health Study Group (2008). Sleep disordered breathing and hypertension: Does self-reported sleepiness modify the association?. Sleep.

[2] Walia HK (2014). Association of severe obstructive sleep apnea and elevated blood pressure despite antihypertensive medication use. J. Clin. Sleep Med..

[3] Qie R (2020). Obstructive sleep apnea and risk of type 2 diabetes mellitus: A systematic review and dose-response meta-analysis of cohort studies. J. Diabetes.

[4] Lim DC, Pack AI (2017). Obstructive sleep apnea: Update and future. Annu. Rev. Med..

[5] Yingjuan M, Siang WH, Alvin TKL, Poh HP (2019). Positional therapy for positional obstructive sleep apnea. Sleep Med. Clin..

[6] Leppänen T (2016). Length of individual apnea events is increased by supine position and modulated by severity of obstructive sleep apnea. Sleep Disord..

[7] Oksenberg A (1997). Positional vs. non-positional obstructive sleep apnea patients. Anthropomorphic, nocturnal polysomnographic and multiple sleep latency test data. Chest.

[8] Heinzer R, Petitpierre NJ, Marti-Soler H, Haba-Rubio J (2018). Prevalence and characteristics of positional sleep apnea in the HypnoLaus population-based cohort. Sleep Med..

[9] Chung JW, Enciso R, Levendowski DJ, Westbrook PR, Clark GT (2010). Patients with positional versus nonpositional obstructive sleep apnea: A retrospective study of risk factors associated with apnea-hypopnea severity. Oral Surg. Oral Med. Oral Pathol. Oral Radiol. Endod..

[10] Ravesloot MJ, van Maanen JP, Dun L, de Vries N (2013). The undervalued potential of positional therapy in position-dependent snoring and obstructive sleep apnea—A review of the literature. Sleep Breath..

[11] Koo BB, Patel SR, Strohl K, Hoffstein V (2008). Rapid eye movement-related sleep-disordered breathing: Influence of age and gender. Chest.

[12] Muraki M (2008). Apnoea–hypopnoea index during rapid eye movement and non-rapid eye movement sleep in obstructive sleep apnoea. J. Int. Med. Res..

[13] Eun YG (2009). Multilevel surgery in patients with rapid eye movement-related obstructive sleep apnea. Otolaryngol. Head Neck Surg..

[14] Petri N (2019). Mandibular advancement device therapy for obstructive sleep apnea: A prospective study on predictors of treatment success. Sleep Med..

[15] Oksenberg A, Kahmaysi I, Silverberg DS, Tarasiuk A (2000). Association of body position with severity of apneic events in patients with severe nonpositional obstructive sleep apnea. Chest.

[16] Haba-Rubio J, Janssens JP, Rochat T, Sforza E (2005). Rapid eye movement-related disordered breathing: Clinical and polysomnographic features. Chest.

[17] Cartwright RD (1984). Effect of sleep position on sleep apnea severity. Sleep.

[18] Sunnergren O, Broström A, Svanborg E (2013). Positional sensitivity as a confounder in diagnosis of severity of obstructive sleep apnea. Sleep Breath..

[19] Pevernagie DA, Shepard JW (1992). Relations between sleep stage, posture and effective nasal CPAP levels in OSA. Sleep.

[20] Oksenberg A (2020). Obstructive sleep apnea: Do positional patients become nonpositional patients with time?. Laryngoscope.

[21] Joosten SA, O'Driscoll DM, Berger PJ, Hamilton GS (2014). Supine position related obstructive sleep apnea in adults: Pathogenesis and treatment. Sleep Med. Rev..

[22] Oksenberg A, Arons E, Nasser K, Vander T, Radwan H (2010). REM-related obstructive sleep apnea: The effect of body position. J. Clin. Sleep Med..

[23] Findley LJ, Wilhoit SC, Suratt PM (1985). Apnea duration and hypoxemia during REM sleep in patients with obstructive sleep apnea. Chest.

[24] Yamauchi M (2015). Nonrapid eye movement-predominant obstructive sleep apnea: Detection and mechanism. J. Clin. Sleep Med..

[25] O'Connor C, Thornley KS, Hanly PJ (2000). Gender differences in the polysomnographic features of obstructive sleep apnea. Am. J. Respir. Crit. Care Med..

[26] Chou YT (2017). Pay attention to treating a subgroup of positional obstructive sleep apnea patients. J. Formos. Med. Assoc..

[27] Dieltjens M (2015). A promising concept of combination therapy for positional obstructive sleep apnea. Sleep Breath..

[28] Campos-Rodríguez F, Fernández-Palacín A, Reyes-Núñez N, Reina-González A (2009). Clinical and polysomnographic features of rapid-eye-movement-specific sleep-disordered breathing. Arch. Bronconeumol..

[29] Trinder J, Kay A, Kleiman J, Dunai J (1997). Gender differences in airway resistance during sleep. J. Appl. Physiol..

[30] Popovic RM, White D (1995). Influence of gender on waking genioglossal electromyogram and upper airway resistance. Am. J. Respir. Crit. Care Med..

[31] Berry RB (2012). Rules for scoring respiratory events in sleep: Update of the 2007 AASM Manual for the Scoring of Sleep and Associated Events. Deliberations of the Sleep Apnea Definitions Task Force of the American Academy of Sleep Medicine. J. Clin. Sleep Med..

[32] Johns MW (1992). Reliability and factor analysis of the Epworth Sleepiness Scale. Sleep.

[33] Kirjavainen M, Kirjavainen T (2007). Upper airway dimensions in class II malocclusion. Effects of headgear treatment. Angle Orthod..

