# Author Correction: Comparison of polysomnographic and cephalometric parameters based on positional and rapid eye movement sleep dependency in obstructive sleep apnea

**DOI:** 10.1038/s41598-023-28471-w

**Published:** 2023-01-20

**Authors:** Jung-Hwan Jo, Sung-Hun Kim, Ji-Hee Jang, Ji-Woon Park, Jin-Woo Chung

**Affiliations:** 1 Department of Oral Medicine, Seoul National University Dental Hospital, 101 Daehak-ro, Jongno-gu, Seoul, 03080 Korea; 2 Department of Oral Medicine and Oral Diagnosis, School of Dentistry and Dental Research Institute, Seoul National University, 101 Daehak-ro, Jongno-gu, Seoul, 03080 Korea

Correction to: *Scientific Reports* https://doi.org/10.1038/s41598-022-13850-6, published online 14 June 2022

The original version of this Article contained an error.

As a result of an administrative mistake the incorrect number of the IRB approval was included in this manuscript. The study was performed under two protocols approved by the Institutional Review Board of Seoul National University Dental Hospital: CRI14037 and CRI20004. As a result, in Methods, under the subheading ‘Subjects’,

“The study was approved by the Institutional Review Board of Seoul National University Dental Hospital and informed consent was obtained from all individual participants included in the study (CRI 08027).”

now reads:

“The study was approved by the Institutional Review Board of Seoul National University Dental Hospital and informed consent was obtained from all individual participants included in the study (CRI14037 and CRI20004).”

The original Article has been corrected.

